# Laparotomy for Advanced Abdominal Ectopic Pregnancy

**DOI:** 10.1155/2022/3177810

**Published:** 2022-03-08

**Authors:** Dereje Tegene, Sultan Nesha, Befikadu Gizaw, Tadele Befikadu

**Affiliations:** Department of Obstetrics and Gynecology, Adama Hospital Medical College, Adama, Ethiopia

## Abstract

**Background:**

Abdominal pregnancy is the rarest and the most serious type of extrauterine pregnancy. The mainstay of treatment for advanced abdominal pregnancy is surgery. The fetus can be delivered easily, and there are two options for the management of the placenta: removal of the placenta and leave the placenta in situ. *Case Presentation*. This is a 26-year-old primigravida lady who does not recall her first day of last normal menstrual period (LNMP) but claimed to be amenorrhic for the past 9 months. She had antenatal care (ANC) follow-up at a private hospital and had obstetric ultrasound two times and told that the pregnancy was normal. Currently, she presented with absent fetal movement of one week and vaginal bleeding of 3 days duration. She had history of abdominal pain with fetal movement before one week. Upon examination, the abdomen was 34 weeks sized, with easily palpable fetal parts; fetal heartbeat was negative, with mild abdominal tenderness. The cervix was closed and uneffaced. She was investigated with ultrasound which reveals 3^rd^ trimester abdominal ectopic pregnancy with negative fetal heartbeat. Laparotomy was done to deliver a 2000 gm female stillborn with GIII maceration from the peritoneal cavity. Placenta was removed after releasing adhesion from the bowel and omentum. She had smooth postoperative course and discharged on her 5^th^ postoperative day.

**Conclusion:**

Abdominal ectopic pregnancy could be missed despite having repeated ultrasound scanning and may continue to third trimester. High index of suspicion and correlation of patient's sign and symptom is very important to make early diagnosis.

## 1. Introduction

Abdominal pregnancy refers to a pregnancy that has implanted in the peritoneal cavity, external to the uterine cavity and fallopian tubes [1]. An abdominal pregnancy is the rarest and the most serious type of extrauterine pregnancy [2]. It accounts 1 to 1.4 percent of all ectopic pregnancies [1, 3]. Early diagnosis of an abdominal pregnancy is difficult since it is associated with a wide range of signs and symptoms [2]. Risk factors for abdominal pregnancy include tubal damage, pelvic inflammatory disease, endometriosis, assisted reproductive techniques, and multiparity [4, 5]. The patient with advanced abdominal pregnancy may present with history of recurrent abdominal discomfort, painful fetal movement beneath the abdominal wall, the presence of fetal movements high in the upper abdomen, cessation of fetal movement, a closed and uneffaced cervix, or the failure of oxytocin to stimulate the gestational mass. Ultrasound is the most effective method for diagnosing an abdominal ectopic pregnancy [2]. The fetus can be delivered easily; the key issue is how to manage the placenta. There are two options for the management of the placenta; the first being removal of the placenta after ligating the placental blood supply, if the placental separation is not difficult [4, 6]. We have to be cautious upon removal of the placenta since it may lead to life-threatening maternal hemorrhage. The second option is leaving the placenta in situ after ligating the umbilical cord [6].

We present a case of abdominal ectopic pregnancy which progressed to third trimester and end up with fetal death. Despite having repeated ultrasound scanning, the diagnosis was missed. It emphasizes the need of high index of suspicion and correlation of patient's sign and symptom with ultrasound finding to make the early diagnosis of abdominal ectopic pregnancy. Removal of the placenta is a preferred option for the placental management, if the placental separation is not difficult. Relevant literatures were also reviewed.

## 2. Case Presentation

This is a 26-year-old primigravida lady who does not recall her first day of last normal menstrual period (LNMP) but claims to be amenorrhic for the past 9 months. She had no early milestones at hand. She had antenatal care (ANC) follow-up at a private hospital in Mojo town. During her ANC follow-up, she had obstetric ultrasound scanning two times, and she was told that the pregnancy was normal. Currently, she was presented with the complaint of absent fetal movement of one week and vaginal bleeding of 3 days duration; the bleeding was bright red and nonclotting. She had history of abdominal pain with fetal movement before one week. Otherwise, she had no history of nausea or vomiting. She was referred to our hospital from Bishoftu General Hospital with the diagnosis of rule out third trimester abdominal ectopic pregnancy after having an obstetric ultrasound, and the reason for referral was for better investigation and management. She was married 10 years back but unable to get pregnant despite having unprotected regular sexual intercourse for the past 10 years. She had no history of pelvic inflammatory disease (PID). She had no history of exposure to ovulation induction drugs or use of assisted reproductive technology. She had no history of chronic medical or surgical illnesses. The pregnancy was planned, wanted, and supported.

**Figure 1:**
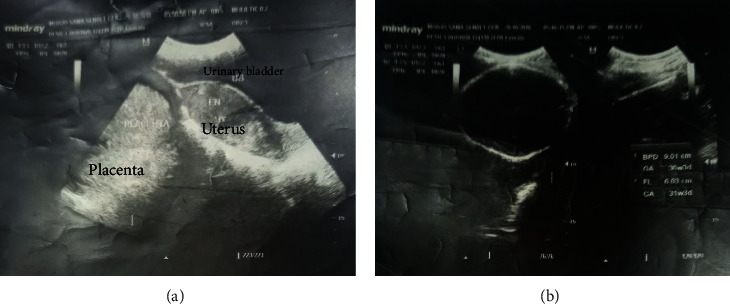
(a and b) Ultrasound findings of abdominal ectopic pregnancy.

Upon physical examination, she was well looking and comfortable, and her vital signs were as follows: blood pressure = 110/70 mmHg, pulse rate = 88 bpm, respiratory rate = 20 breaths per minute, and temperature = afebrile to touch. She had pink conjunctive and nonicteric sclera. Pertinent findings were on the abdomen: the abdomen was 34 weeks sized with irregular outline and easily palpable fetal parts, fetal heartbeat (FHB) was negative, with mild abdominal tenderness, and there was no signs of fluid collection. On genitourinary system (GUS), she had normal external female genitalia, she had no active vaginal bleeding, the cervix wa[1, 7]s closed and uneffaced, there are no cervical motion tenderness and palpable adnexal mass, and upon bimanual examination, the cervix does not move with the fetal parts. She was investigated with obstetric ultrasound and reported as follows: she had singleton extrauterine pregnancy, with an empty uterus measuring 9 cm∗10 cm, FHB was negative, the placenta was attached to the posterior abdominal wall near to the blood vessels, and gestational age (GA) by femur length (FL) was 31 weeks + 3 days. The index was 3^rd^ trimester abdominal ectopic pregnancy with fetal death (Figures 1(a) and 1(b)). Her laboratory investigation reveals the following: white blood cell = 6,290/mm^3^; hemoglobin = 11.5 g/dl; platelet count =404 × 10^3^/ml, and blood group and Rh = O^+^. Coagulation profile shows the following: PT = 15 seconds, aPTT = 28.7 seconds, and INR = 1 ratio.

**Figure 2:**
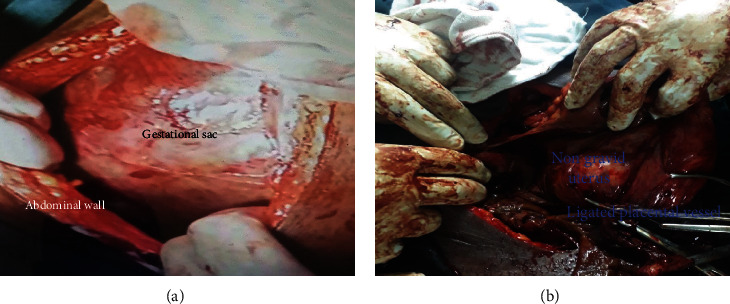
(a and b) Intraoperative findings showing gestational sac with the peritoneal cavity, nongravid sized uterus, and meconium-stained bowel.

With the diagnosis of primigravida + 3^rd^ trimester abdominal ectopic pregnancy + fetal death, the plan was to admit her to the ward and to have abdominopelvic MRI to confirm the diagnosis and to assess the placental attachment with the major vessels. Since MRI was not available in our hospital and the patient could not afford the cost at a private facility, MRI was not done. The surgical team was consulted, and she was evaluated by the team. After the discussion with the surgical team, decision for laparotomy was made. After informed written consent was obtained from the patient and 4 unit of cross-matched blood was prepared, the patient was taken to the operation theater. Under general anesthesia, the abdomen was entered through the midline incision. Intraoperative findings were as follows: There was a large gestational sac containing well-formed fetus and placenta within the peritoneal cavity. The sac was adherent to the cecum and mesentery of gastrointestinal tract. There was no adhesion of the placenta with the major organs and major vessels. There was an intact 8-week-sized nongravid uterus with healthy looking left ovaries and tube. The right ovary and tube were not identified due to dense adhesion (Figures 2(a) and 2(b)).

**Figure 3:**
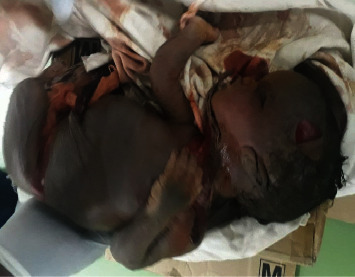
A 2000 gm female stillborn with grade-III maceration delivered from the peritoneal cavity.

What was done was after opening the sac, a 2000 gm female stillborn with GIII maceration was delivered (Figure 3). Meconium-stained amniotic fluid was sucked from the sac. Adhesion with the bowel and omentum was released by sharp dissection, and the placenta was taken out from the peritoneal cavity. After securing hemostasis, exploration of the bowel was made for possible bowel injury. The peritoneal cavity was lavaged with 4 liter of normal saline, and the abdomen was closed in layer. Estimated blood loss was 700 ml. The patient transferred to the ward with stable vital sign and postoperative orders. Postoperatively, the patient was on maintenance fluid, parenteral antibiotics, and standing dose of analgesia. She had smooth postoperative course, and her post-op hemoglobin was 9.4 g/dl. She was discharged on her 5^th^ postoperative day with therapeutic dose of ferrous sulfate and appointed after 1 week. She was seen at an outpatient clinic three times and she had no new compliant.

## 3. Discussion

An ectopic pregnancy is a pregnancy where the blastocyst implants anywhere other than the endometrial cavity and estimated to occur in 1 to 2% of all pregnancies . Nearly 95% of ectopic pregnancies implant in the fallopian tube, while the remaining implant in other locations such as the abdomen, cesarean scar, cervix, and ovary [1]. Abdominal pregnancy refers to a pregnancy that has implanted in the peritoneal cavity, external to the uterine cavity and fallopian tubes [1]. An abdominal pregnancy is the rarest and the most serious type of extrauterine pregnancy. The reported incidence of abdominal pregnancy varies, ranging from 1 in 3,371 deliveries to 1 in 10,000 deliveries [2]. It accounts 1 to 1.4 percent of all ectopic pregnancies [1, 3].

Abdominal pregnancies are classified as primary or secondary [2]. Primary abdominal pregnancy is a result of intra-abdominal fertilization of sperm and ovum, with primary implantation in the abdomen [3, 6]. A secondary abdominal pregnancy is the most common type and is the result of early tubal abortion or rupture with secondary implantation of the pregnancy into the peritoneal cavity [2]. Rare types of secondary abdominal pregnancies have occurred after spontaneous separation of an old cesarean section scar, after uterine perforation during a therapeutic or elective abortion, and after subtotal or total hysterectomy [2]. Potential sites of abdominal ectopic pregnancy include the omentum, pelvic sidewall, broad ligament, posterior cul-de-sac, abdominal organs (e.g., spleen, bowel, and liver), large pelvic vessels, diaphragm, and uterine serosa [6, 8]. The pouch of Douglas (POD) is the most common location of abdominal pregnancy followed by the mesosalpinx and omentum [8].

Early diagnosis of an abdominal pregnancy is difficult since it is associated with a wide range of signs and symptoms [2]. In a case series of 10 abdominal pregnancies, only 6/10 were diagnosed preoperatively [9]. A high index of suspicion is important for making a diagnosis of abdominal pregnancy. Risk factors for abdominal pregnancy include tubal damage, pelvic inflammatory disease, endometriosis, assisted reproductive techniques, and multiparity [4, 5].

In contrast to tubal ectopic pregnancies, abdominal pregnancies may go undetectable until an advanced gestational age. In such cases, the patient may present with history of recurrent abdominal discomfort, painful fetal movement beneath the abdominal wall, the presence of fetal movements high in the upper abdomen, cessation of fetal movement, a closed and uneffaced cervix, or the failure of oxytocin to stimulate the gestational mass, and the fetus may assume an unusual lie [2]. Nausea and vomiting may be prominent symptoms, when the pregnancy implants on bowel. Compared with tubal ectopic pregnancies, vaginal bleeding is less frequent in abdominal ectopic pregnancy; however, vaginal bleeding may occur since the endometrium still responds to hormonal changes of pregnancy [10].

Ultrasound is the most effective method for diagnosing an abdominal ectopic pregnancy and can usually identify an abdominal gestation as separate from the nonpregnant uterus [2]. Ultrasound, especially transvaginal, remains the first-line tool for diagnosing abdominal pregnancy [11]. The classic ultrasound finding is the absence of myometrial tissue between the maternal bladder and the pregnancy [6]. An empty uterus may be visualized. Other findings include poor definition of the placenta, oligohydramnios, and unusual fetal lie. An advanced abdominal gestation may be misinterpreted as being intrauterine if the ultrasonographer does not evaluate the myometrium during the examination [12]. CT scan and MRI can be useful for confirming the diagnosis, distinguishing anatomic relationships and potential vascular connections, and assessing placental adherence [13].

Abdominal pregnancies, even when advanced, are interrupted at diagnosis, as the potential for delivery of a healthy infant is poor and the risk of maternal complications is high. If the diagnosis is made late in pregnancy, a viable infant may be delivered via laparotomy. Expectant management to gain fetal maturity has been attempted and has been successful in a few cases [14]. The mainstay of treatment of advanced abdominal pregnancy is surgery, but the optimal approach has not been determined. The fetus can be delivered easily; the key issue is how to manage the placenta. There are two options for the management of the placenta: the first being removal of the placenta after ligating the placental blood supply, if the placental separation is not difficult [4, 10]. We have to be cautious upon removal of the placenta since it may lead to life-threatening maternal hemorrhage. The second option is leaving the placenta in situ after ligating the umbilical cord. The patient can then be followed without further intervention, or active intervention using arterial embolization or methotrexate can be instituted to hasten involution [6]. This is a preferred option when the placenta cannot be easily separated. Nonetheless, this approach has its own complications including abscess formation, sepsis, and delayed hemorrhage [15]. Preoperative imaging with MRI has been used successfully to determine the location and attachment of the placenta to aid in this decision [16].

The risk of maternal death from abdominal pregnancy is 7.7 times greater than the risk of maternal death from tubal ectopic pregnancy and 90 times greater than that with intrauterine pregnancy. Reported maternal mortality rates in the literature have varied in the past from 4% to 29% [2]. Maternal death is usually the result of uncontrollable hemorrhage [17]. Maternal morbidity can also be substantial, with high incidences of pelvic abscess, peritonitis, and sepsis caused by retained placental remnants. Rare instances of massive rectal bleeding or rectal passage of fetal bones secondary to the formation of celo-intestinal fistula have also been reported. Fetal mortality is notoriously high, ranging from 75% to 95% of all cases [2]. Fetal deformations and perinatal death occur more often than maternal death [17]. Common fetal abnormalities include facial and/or cranial asymmetry, joint abnormalities, hypoplastic limbs, and central nervous system malformations. Pregnancies with some vascular attachment to the uterus seem to be associated with a higher chance of fetal survival [6].

In this case, she had no identified risk factors for abdominal ectopic pregnancy. She is a primigravida with no history of PID and no exposure to assisted reproductive technology. Clinically, she had symptom suggestive of advanced abdominal ectopic pregnancy; this includes abdominal pain with fetal movement, cessation of fetal movement of 1 week duration, and vaginal bleeding of 3 days duration. Even though it is not common as tubal ectopic pregnancy, vaginal bleeding may occur in abdominal ectopic pregnancy; this is due to the endometrial response to hormonal changes of pregnancy [10]. Easily palpable fetal parts and closed and uneffaced cervix are also suggestive physical findings of abdominal ectopic pregnancy. The fetus was dead. The fetal mortality rate is very high in advanced abdominal ectopic pregnancy, reported as 75% to 95% [2]. Although ultrasound is the most effective method for the diagnosis of abdominal ectopic pregnancy, it was missed twice in our case. This can be due to the lack of experience of the radiographer and inability to evaluate the myometrium at the time of scanning. Since the placental separation was not difficult, the placenta was removed after ligating the placental vessels. There was no intra-abdominal hemorrhage. She was followed at an outpatient clinic for 3 months, and she had no complication.

## 4. Conclusion

This is an abdominal ectopic pregnancy in a 26-year-old woman who attempted to be pregnant for 10 years, and the pregnancy progressed to third trimester and end up with fetal death. Abdominal ectopic pregnancy could be missed despite having repeated ultrasound scanning and may continue to third trimester. High index of suspicion and correlation of patient's sign and symptom with ultrasound findings is very important to make the early diagnosis of abdominal ectopic pregnancy. Removal of the placenta is a preferred option for the placental management, if the placental separation was not difficult.

## Data Availability

All data generated in the preparation of this document are included in the submitted manuscript, and the datasets used in this case report can be obtained from the corresponding author.

## Abbreviations

AHMC: Adama Hospital Medical College
ANC: Antenatal care
FHB: Fetal heartbeat
GA: Gestational age
IUFD: Intrauterine fetal death
MRI: Magnetic resonance imaging.
